# Developing Immune Profiles of Endangered Australian Sea Lion (*Neophoca cinerea*) Pups Within the Context of Endemic Hookworm (*Uncinaria sanguinis*) Infection

**DOI:** 10.3389/fvets.2022.824584

**Published:** 2022-04-21

**Authors:** María-Ignacia Meza Cerda, Rachael Gray, Peter C. Thomson, Loreena Butcher, Kelly Simpson, Abby Cameron, Alan D. Marcus, Damien P. Higgins

**Affiliations:** Faculty of Science, Sydney School of Veterinary Science, The University of Sydney, Sydney, NSW, Australia

**Keywords:** Australian sea lion, ecoimmunology, hookworm, acute-phase proteins, IgG, PCR

## Abstract

As a top predator, the endangered Australian sea lion (*Neophoca cinerea*) is a sentinel of ecosystem change, where population trends can reflect broader shifts in the marine environment. The population of this endemic pinniped was historically diminished by commercial sealing, and recovery has been slowed by fishery interactions, disease and, potentially, pollutants. Hookworm infects 100% of neonatal pups and has been identified as a contributor to population decline. Here, a multivariable approach using traditional serological and novel molecular tools such as qPCR and ddPCR was used to examine immune phenotypes of developing Australian sea lion pups infected with the endemic hookworm (*Uncinaria sanguinis*) from two South Australian colonies. Results show changing immunophenotypes throughout the patent period of infection represented by pro-inflammatory cytokines (IL-6), IgG and acute-phase proteins. Although cytokines may prove useful as markers of resistance, in this study, IL-6 is determined to be an early biomarker of inflammation in Australian sea lion pups, excluding the alternative hypothesis. Additionally, immunological differences between animals from high- and low-intensity hookworm seasons, as well as ivermectin-treated animals, indicate hookworm infection modulation of the host immune response, as evidenced by a lower IL-6 mRNA expression in the non-treated groups. This study of the Australian sea lion is an example of an ecoimmunological approach to disease investigation, which can be applied to evaluate the impact of environmental and anthropogenic factors on susceptibility to infectious diseases in free-ranging species

## Introduction

Infectious wildlife disease, emerging or endemic, can play a beneficial or neutral role as a component of species ecology or can have detrimental impacts, either directly or as an additive pressure or amplifier of other threats. As such, understanding mechanisms of disease susceptibility can inform conservation management of threatened species (1, 2). Measuring variation in innate and adaptive immune parameters offers an additional perspective for health assessment and evaluation of susceptibility to environmental pressures in wildlife populations (3). For example, impaired innate and humoral immune responses have been reported in California sea lions (*Zalophus californianus*) exposed to climatic abnormalities (4) and Galapagos sea lions (*Zalophus wollebaeki*) exposed to anthropogenic stressors (5). The ecoimmunology approach used in these and similar studies has emerged as a valuable means to assess immunocompetence in wild populations in the context of diseases, pollution or environmental threats (6, 7).

Understanding host-pathogen relationships and the environmental factors that influence them is fundamental to predicting and managing disease outcomes in animals (6, 8). The Australian sea lion (*Neophoca cinerea*) is endemic, listed as “Endangered” under the IUCN and the Australian Environment Protection and Biodiversity Conservation Act 1999 (9, 10), and experiences high pup mortality rates largely associated with conspecific trauma and hookworm infection (*Uncinaria sanguinis*) (11, 12). Two aspects of hookworm disease in Australian sea lions suggest that changing environmental factors could predispose animals to disease. Firstly, the host-pathogen relationship is longstanding (13, 14), and although the historical impact of this disease is unknown, its current prevalence and impact are currently high. Secondly, disease outcomes vary markedly among individuals despite a 100% prevalence of infection (15). In the South American fur seal (*Arctocephalus australis*), climate conditions influenced the cellular and humoral immune response to hookworm (*Uncinaria* sp.) infection in pups (16). It is also possible that host traits such as sex, genetics and age play a role as sources of variation on the host immune response (17). For example, wild male wood mice (*Apodemus sylvaticus*) have higher local and systemic TNFα expression during parasitic infections compared to females (18). In California sea lions, heterozygosity was described to be a helpful predictor of the immune response against hookworm infection (19). Although the epidemiology, clinical evaluation and impact of hookworm infection have been described in two of the largest Australian sea lion colonies in South Australia (Seal Bay and Dangerous Reef) (11, 15, 20), the immunological host responses and the factors that can influence them are not yet understood.

Immunological studies to understand drivers of disease in free-range Australian sea lions could prove helpful for the conservation management of populations. For example, understanding the effect of ecotoxicological biomarkers recently described for Australian pinnipeds (21, 22) on health and immunity could inform management regulations. Immunological studies have highlighted the role of pollutants, tourism and co-infections as stressors for Galapagos sea lions (5) and bottlenose dolphins (23, 24) and contributed toward the informed design of management actions (23–25). However, in free-ranging Australian sea lion pups, our understanding of immunity is limited to the description of a lymphocytic-eosinophilic response with hypoproteinemia in association with hookworm (11). Immunological mechanisms associated with hookworm infections have been mostly inferred from humans [Reviewed in (26)], mouse and hamster models (27–29), dogs (30) and other otariids (16).

Age and ontogeny must be considered when assessing the impact of environmental factors and disease on immune systems. Stage of development influences immune function in young mammals, suggesting periods of greater susceptibility to infections (31–33). For example, in pinniped pups, passive immunity acquired through the transfer of maternal antibodies *in utero* or through the ingestion of colostrum, occurs in lower rates compared to other mammals (34–36). In this way, the lower plasma IgG concentrations found in New Zealand sea lion (*Phocarctos hookeri*) pups <2 weeks of age, compared to older animals, could mean greater vulnerability to bacterial infections such as *Klebsiella pneumoniae* (37), until their innate immunity starts to develop. Similar age dependencies in serum protein profiles have been reported in bovine (*Bos taurus*) calves (38) and dogs (*Canis familiaris*) from 6 weeks to adulthood (39). The development of protective immune responses is highly plastic and depends largely on the cytokine milieu, the pathogen load at a specific point in time, and the naïve immune system at the early stages of life (40). Ideally, these patterns would be understood before evaluating the impacts of environmental and pathogen factors on immune function. However, this is impractical for most species and populations, in particular endangered species with limited captive populations.

Using a multifactorial approach is essential when examining immune impacts and disease in general, particularly when studying heterogeneous populations of relatively unstudied animals in complex environments. The immune system is a complex network where components of innate and adaptive immunity interact to provide hosts with a protective response (41, 42). Reductionist approaches from previous decades, imposed mainly by technological restrictions (3, 43), have therefore evolved into more complex study models involving multiple approaches and parameters, such as longitudinal sampling in wild animals and a range of molecular phenotypic measures (44, 45). Functional assays applicable across species, such as lysozyme activity, acute phase proteins (APP) or cutaneous responses to phytohaemagglutinin (PHA) mitogen, have been supplemented more recently by the significant advances made in the area of molecular immunology, reducing the implementation costs of species-specific immune assays and expanding the ecoimmunologists' tool kit (46–48). Depending upon the target chosen, cytokine gene expression can be used to quantify upregulation of pro-inflammatory cytokines from activation of the innate response (IL-1, TNFα, IL-6) (49, 50) and subsequent activation of antigen-presenting cells (APC), or those involved in modulation of the adaptive response toward T-helper 1 (Th1, IFNγ; cellular responses to intracellular pathogens) or T-helper 2 (Th2, IL-4; humoral responses to extracellular pathogens, parasites and repair) pathways (51, 52). For example, pro-inflammatory cytokines, such as IL-1, IL-6, and IL-12, and APP were found in higher concentrations in harbor seal (*Phoca vitulina*) pups in the early period of rehabilitation, with IL-4 dominance occurring at the later stages (49). Interleukin 10 expression has been associated with chronic bacterial infections in harbor porpoises (*Phocoena phocoena*) (53). Utilizing these novel approaches and knowledge will enable a more comprehensive understanding of immune phenotypes and how they relate to the dynamics of parasitic diseases in threatened wildlife populations such as the Australian sea lion.

In this study, we examine immune phenotypes of neonatal Australian sea lion pups sampled at two South Australian colonies, Seal Bay and Dangerous Reef, where infection with hookworm is endemic. Previous studies have determined the intensity of hookworm infection in pups found dead during summer and winter breeding seasons at both colonies, identifying colony-specific associations between hookworm infection intensity and season (i.e., summer associated with high intensity at Seal Bay and low intensity at Dangerous Reef) (11). Clinical parameters of health and hookworm infection status (11, 54) are combined with measures of the innate and adaptive immune response, including total IgG, lysozyme activity and constitutive gene expression of cytokines IFNγ, IL-6, IL-10 and TNFα in order to characterize the immunological response to hookworm infection in Australian sea lion pups, and to identify developmental trends in immune parameters. To identify relevant markers, immune parameters of non-treated cohorts of pups from Seal Bay (high-intensity hookworm season) and Dangerous Reef (low-intensity hookworm season) and anthelmintic (ivermectin) treated pups from Dangerous Reef are compared. We hypothesize that innate and adaptive immunity will vary with (1) the developmental age of pups, (2) differing hookworm infection intensity seasons (colonies), and (3) between non-treated and an anthelmintic-treated cohort of pups sampled at Dangerous Reef.

## Materials and Methods

### Study Area and Sample Collection

Samples were collected from Australian sea lion pups during summer breeding seasons at Seal Bay (35.994° S, 137.317° E) in 2012 (high-intensity hookworm season) and Dangerous Reef (34.815° S, 136.212° E) in 2013 (low-intensity hookworm season), two of the largest and biogeographically diverse Australian sea lion colonies. At both colonies, the endemic occurrence of *U. sanguini*s in neonatal pups is 100 % (15). Veterinary observations and assessments recorded at the time of sample collection did not indicate any clinical suspicion for the presence of significant systemic microbiological or other infectious disease (Gray, pers. Comm.). Fecal smears and formalin-fixed intestinal contents were microscopically examined, which, together with postmortem (gross necropsy) observations, ruled out the presence of other macroparasites (15). A subset of pups sampled at multiple time points by Marcus et al. (11) was selected for this study using the following criteria: pups had patent hookworm infection at first capture (hookworm eggs identified in fecal smears) and the following were available for analyses from each time point—complete hematological and host parameters (i.e., age and/or standard length, sex and disease severity), archived serum and plasma samples; archived blood samples stored in FACS lysing solution; and archived RNA samples. Detailed methods for pup handling and sample collection are given in Marcus et al. (11). From Seal Bay, a cohort of known-age pups during the patent and post-patent period of hookworm infection was selected, including *n* = 7 pups with three capture events 2–4 weeks apart and *n* = 16 pups with two capture events 3–5 weeks apart. The Dangerous Reef cohort included pups of unknown age with patent hookworm infection that were likely to be <2 months old (standard length <70 cm and non-molting status) (15). From sampling at Dangerous Reef, *n* = 18 pups were selected with two capture events 4–5 weeks apart (54). Of these, a subset of pups (*n* = 8) were administered 200 μg/kg ivermectin by subcutaneous injection in the dorsal interscapular region (10 mg/mL IVOMEC Antiparasitic Injection for Cattle, Merial Australia, Sydney, Australia), resulting in negative hookworm status (no eggs on fecal smears based on examination of a subsequent sample). Control pups (*n* = 10) were administered 0.02 mL/kg saline (0.9 % sodium chloride, Baxter Healthcare, Sydney, Australia), subcutaneously in the dorsal interscapular region (54). For statistical analyses, three treatment groups were defined: DR treatment (*n* = 8), DR (low-intensity) control (pups administered placebo; *n* = 10) and SB (high-intensity) control (*n* = 23).

The hematological data from Marcus et al. (11) used in this study were: packed cell volume (PCV), total plasma protein (TPP), and absolute corrected white blood cell (cWBC), absolute lymphocyte (Lymph), and eosinophil (Eos) counts. Host factors included standard length (cm), sex and disease severity. Age was recorded only for pups at Seal Bay (*n* = 23) (15); therefore, standard length was used as a proxy for age when analyzing data from both colonies (55). As eggs per gram of feces is not a reliable measure of hookworm load (15), the severity of hookworm disease in pups was classified as “mild” (TPP ≥ 60 g/L and PCV > 35%) or “severe” (TPP < 60 g/L and PCV ≤ 35%) based on descriptions of hookworm infection outcomes in humans (hypoproteinemia and anemia) (56) and hematological values for Australian sea lions (11, 57).

### Sample Preparation and Storage

Blood samples (89 samples from 41 individuals) were collected from the brachial vein of *N. cinerea* pups and then transferred to Ethylene diamine tetra-acetic acid (EDTA) and plain serum tubes (Sarstedt, Nümbrecht, Germany). For complete hematological analysis, EDTA anti-coagulated whole blood samples were stored at 4°C and processed within 10 hours of collection (11). In the field, plasma and serum samples for IgG ELISA, serum protein electrophoresis (SPE) and lysozyme assays were frozen in cryotubes in a liquid nitrogen dry shipper and later transferred to a −80°C freezer for long term storage until analysis. Serum protein electrophoresis was performed only on pup samples collected at Seal Bay for which age was known (*n* = 23 individuals; seven animals with three capture events and 16 with two capture events).

For flow cytometry, 150 μL of whole blood was placed in 1 × FACS lysing solution (BD Biosciences, San Jose, USA) at a ratio of 1:10 and held at room temperature in the dark for 15 min to lyse red blood cells and fix peripheral blood leukocytes. Following this, samples were stored in liquid nitrogen and later transferred to a −80°C freezer for long-term storage until flow cytometry protocol was performed. Analysis of lymphocyte subsets by flow cytometry (*n* = 15 individuals) included six animals with three capture events and four with two capture events from Seal Bay, and five animals with two capture events from Dangerous Reef.

Aliquots (0.5 mL) of EDTA anti-coagulated whole blood samples were centrifuged at 5,000 x g for up to 3 min. Plasma was removed using a sterile disposable pipette and the remaining red blood cells and buffy coat were resuspended in 1,300 μl RNAlater™ (Applied Biosystems, Carlsbad, CA, USA), stored at 4°C for 2–4 days and then at −20°C until RNA extraction for gene expression assays was performed. Gene expression analysis (*n* = 33 individuals) was undertaken in samples collected from six pups with three capture events and ten pups with two capture events from Seal Bay and from 17 pups with two capture events from Dangerous Reef. The integrity of isolated RNA was demonstrated in all blood samples by the amplification of GAPDH mRNA (Ct 25 ± 3.1, mean ± SD).

The immune parameters used in this study (i.e., constitutive gene expression of cytokines IFNγ, IL-6, IL-10, and TNFα) were selected based on their role as markers of key immunological pathways. Specifics of the laboratory methods used in this study for the quantification of immune measures are presented in Appendix A. Table 1 summarizes the immune variables and methods used in this study.

**Table 1:** Summary of samples size (*n*) and immune measures used in this study.

**Colony**	**Treatment group**	**N**°**of pups sampled (*n*)**	**Capture events**	**Immune Assays**	**References**
Seal Bay	Control	7	3 (total of 21 time points)	IgG ELISA, SPE, Lysozyme assay, Flow Cytometry (T and B lymphocytes), RT-qPCR and ddPCR	Osserman and Lawlor (58), Bossart et al. (59), Hall et al. (60), Gray et al. (61), Lau et al. (62), Meza Cerda et al. (48)
		16	2 (total of 32 time points)		
Dangerous Reef	Control	10	2 (total of 20 time points)	IgG ELISA, Lysozyme assay, Flow Cytometry (T and B lymphocytes), RT-qPCR and ddPCR	
	Ivermectin	8	2 (total of 16 time points)		
Total		41	89		

### Statistical Analysis

#### Preliminary Analysis and Principal Component Analysis (PCA)

An exploratory analysis was initially performed to examine data distributions, identify outliers and visualize trends in the data. Logarithmic data transformations were applied and retained if improvements in normality were observed in histograms. Associations of immune parameters with categorical variables such as sex, treatment group (DR treatment, DR control and SB control) and disease severity (mild and severe) were explored using side by side boxplots and associations with continuous variables were examined using bivariate scatter plots and Pearson's correlations. Significant relationships between continuous variables and collinearity were considered for the subsequent multivariable analyses and outliers were retained if no substantially different models were produced when excluding them.

Principal component analysis on the correlation matrix was used to explore associations between immune parameters in the multivariate dataset and to link sets of functionally related variables. Specifically, clusters related to the three treatment groups were expected to test the hypothesis that hookworm infection can modulate the immune response of pups. Principal component analysis included the following immunological measurements for which complete sample sets for an individual pup were available: IgG, lysozyme, delta cycle threshold (ΔCt) IL-6, ΔCt IL-10, ΔCt TNFα, IFNγ (copy number), and health parameters: PCV, TPP and absolute cWBC, lymphocyte and eosinophil counts. A linear model was used to assess the significance of identified clusters in explaining the observed variance structure of the immunological variables identified in the PCA.

All analyses were conducted using R software version 4.0.0 (63).

#### Multivariable Analyses for Host and Environmental Variables

Following PCA, the hypothesis that immune-related changes are associated with age and that hookworm infection can modulate the immune response of pups was subsequently tested by fitting models on immune variables. Linear mixed models (LMMs) were used to analyse variation in individual immunological measurements using treatment group, sex, disease severity, standard length (proxy for age) and all possible two-way interactions as fixed effects to model environmental and host variables. To address the correlation introduced by repeated measures obtained from the same individual, random effects were included for each pup. Flow cytometry and SPE multivariable analyses were restricted to fewer fixed effects (i.e., age, sex and disease severity for SPE; standard length, treatment group and disease severity for flow cytometry) due to the smaller sample size. The package lme4 in R software was used to fit LMMs, and spline terms were included to fit predictors with possible non-linear relationships to the response variables (such as response evolving over time) (64). All models were constructed using a backwards stepwise procedure until all remaining variables had a *p*-value of < 0.1. In the final model, variables with *p* ≤ 0.1 were considered suggestive of associations and variables with *p* < 0.05 were considered significantly associated with the outcome variable. The Akaike information criterion (AIC) was used to distinguish among a set of possible models describing the relationship between explanatory variables (65). Fitted value plots and histograms of residuals were used to visually assess the assumptions of homogeneity of residual variance and normality of final models. Where necessary, the data were log-transformed. *Post-hoc* pairwise comparisons between factor levels were made using Tukey's test (66).

## Results

### Principal Component Analysis

**Figure 1:**
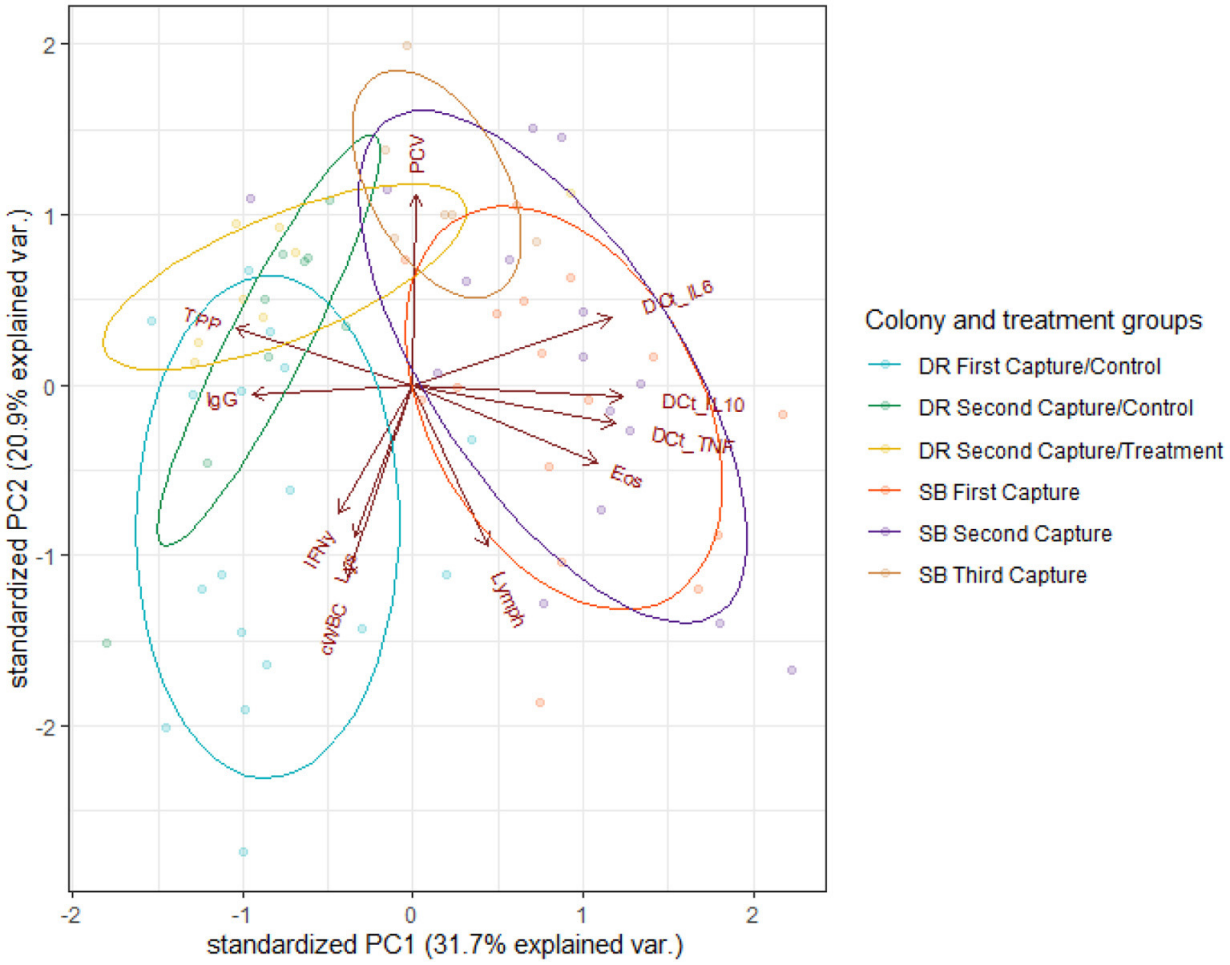
Biplot of immunological and health parameters in Australian sea lion pups (*N. cinerea*) from Dangerous Reef (DR) First Capture-Control (blue ellipse), Dangerous Reef Second Capture-Control (green ellipse), Dangerous Reef Second Capture-Treatment (yellow ellipse), Seal Bay (SB) First Capture (red ellipse), Seal Bay Second Capture (purple ellipse) and Seal Bay Third Capture (brown ellipse). Arrows display PCA loading vectors with the arrow's length and direction, indicating strength and increasing variance. Angles between arrows are representative of the strength of the correlation between variables, with small angles showing highly correlated variables. The dots represent individual pup samples. PCV, Pack cell volume; TPP, Total plasma protein; IgG, Immunoglobulin G; Lys, Lysozyme; cWBC, White blood cells count; Lymph, Lymphocytes count; Eos, Eosinophils count; IFNγ, Interferon-γ; ΔCt IL-6, ΔCt IL-10, ΔCt TNFα.

Based on PCA analysis, the first two principal components (PC1, PC2) explained 52.6% of the variance in the data. PC1 accounted for 31.7% of the samples' variance, while PC2 accounted for 21.9%. Figure 1 shows a biplot of the scores for the first two PCs, as well as clusters identifying the treatment groups. Relationships among parameters of immune function (as shown by vectors on the biplot) were consistent among treatment groups. PC1 is comprised of large positive loadings for ΔCt IL-6, ΔCt IL-10, ΔCt TNFα and absolute eosinophil count, and large negative loadings for TPP and IgG. Likewise, PC2 comprised large positive loadings for PCV and large negative loadings for cWBC, absolute lymphocyte count, lysozyme and IFNγ copy numbers. High PC1 scores separated Seal Bay (high-intensity season) from Dangerous Reef (low-intensity season) individuals. Ivermectin-treated pups from Dangerous Reef, although clustered together, overlapped with the control group, such that there was no appreciable effect of treatment on pups sampled at Dangerous Reef (Figure 1).

Colony / hookworm intensity season explained 62% of the total variance in PC1 [*F*_(2,69)_ = 57.47, *p* < 0.001] with a significant difference [*t*_(69)_ = 10.09, *p* < 0.001] in PC1 scores when the SB control and the DR control group were compared. There was no significant difference seen in the comparison of the DR treatment and the DR control groups [*t*_(69)_ = 0.40, *p* = 0.69] when individual levels of the predictor (DR treatment, DR control and SB control) were further evaluated (Figure 1).

### Multivariable Analysis for Host and Environmental Variables

**Figure 2:**
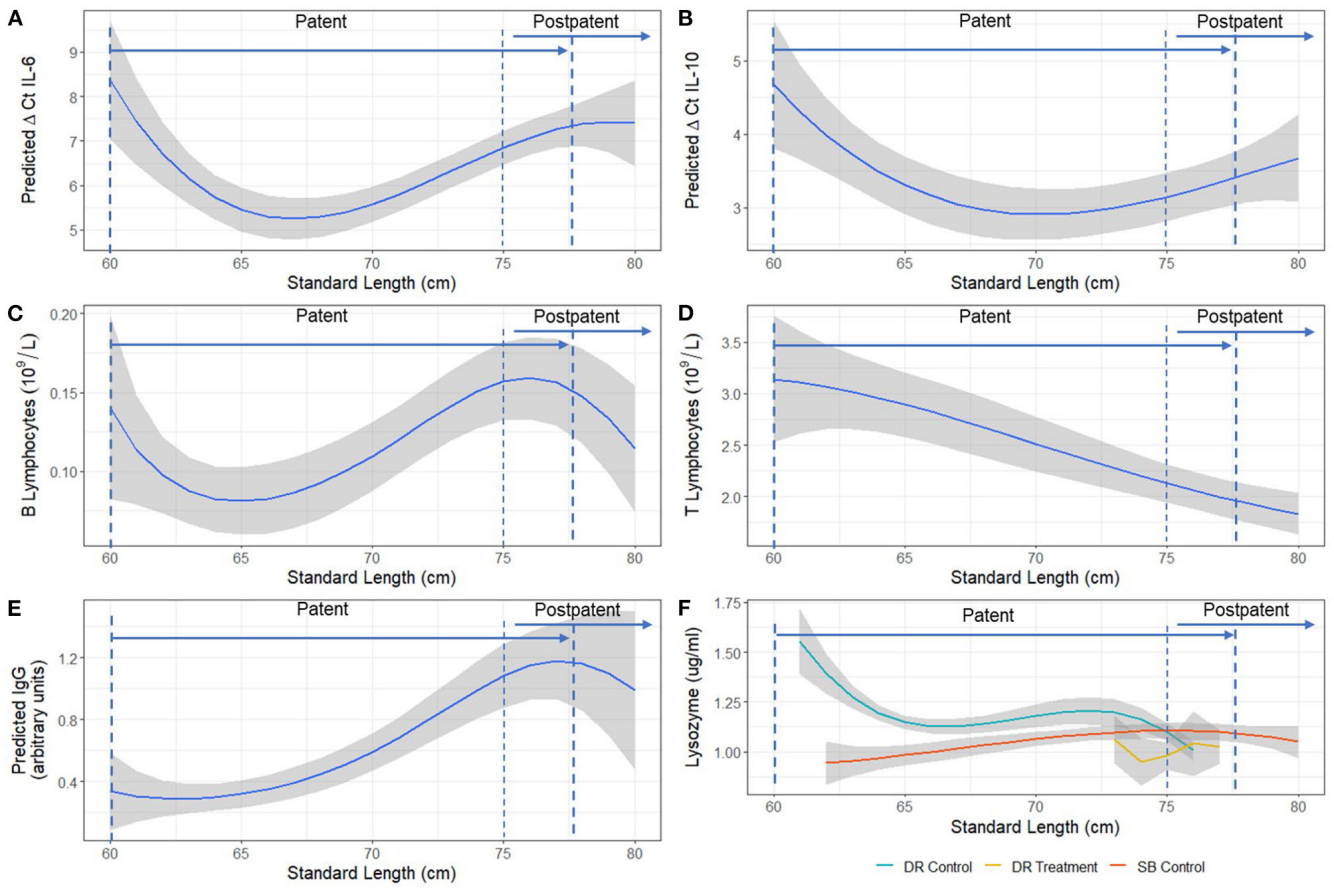
Model estimated relationship between **(A)** ΔCt IL-6, **(B)** ΔCt IL-10, **(C)** B lymphocytes, **(D)** T lymphocytes, **(E)** IgG, and **(F)** Lysozyme and standard length (cm) of Australian sea lion (*N. cinerea*) pups. Statistical models for T and B lymphocyte populations and IgG were performed on a log scale, but model-derived back-transformed means (emmeans package R) are presented. The shaded area represents the 95% confidence interval. Lower ΔCt indicates greater cytokine expression. Patency periods adapted from Marcus et al. (15).

Temporal changes in ΔCt IL-6, ΔCt IL-10, derived B and T lymphocyte numbers, IgG, and lysozyme (Figure 2) were non-linear. Significant length-related changes were seen for gene expression of IL-6 [*F*_(3,54)_ = 4.55, *p* < 0.01] and IL-10 [*F*_(3,33)_ = 3.93, *p* < 0.01], and derived T lymphocytes [*F*_(3,32)_ = 4.36, *p* = 0.01], with an overall decreasing trend with pup age (Figures 2A,B,D). A similar but opposite trend and significant correlation with length was evidenced for B lymphocytes [*F*_(3,30)_ = 2.59, *p* < 0.05] after adjusting for the significant effect of treatment group [*F*_(2,30)_ = 10.80, *p* < 0.001] (Figure 2C), and for IgG [*F*_(3,59)_ = 3.13, *p* < 0.05], after adjusting for the non-significant effect of ivermectin treatment and the significant effect of sex [*F*_(3,59)_ = 4.47, *p* = 0.038] (Figure 2E). The expression of TNFα was not correlated with increasing pup age, but both TNFα and IL-6 models showed a significant positive correlation with each other [*F*_(1,49)_ = 47.70, *p* < 0.001 and *F*_(1,61)_ = 10.1630, *p* = 0.002, respectively]. Overall, control pups from Seal Bay and Dangerous Reef showed a decrease in the expression of IL-6 (that is, higher ΔCt values) followed by a uniform increase in B lymphocytes and relative IgG concentration over time, arriving to a peak coinciding with a standard length of 75 ± 5 cm (60 ± 5 days estimated age).

**Figure 3:**
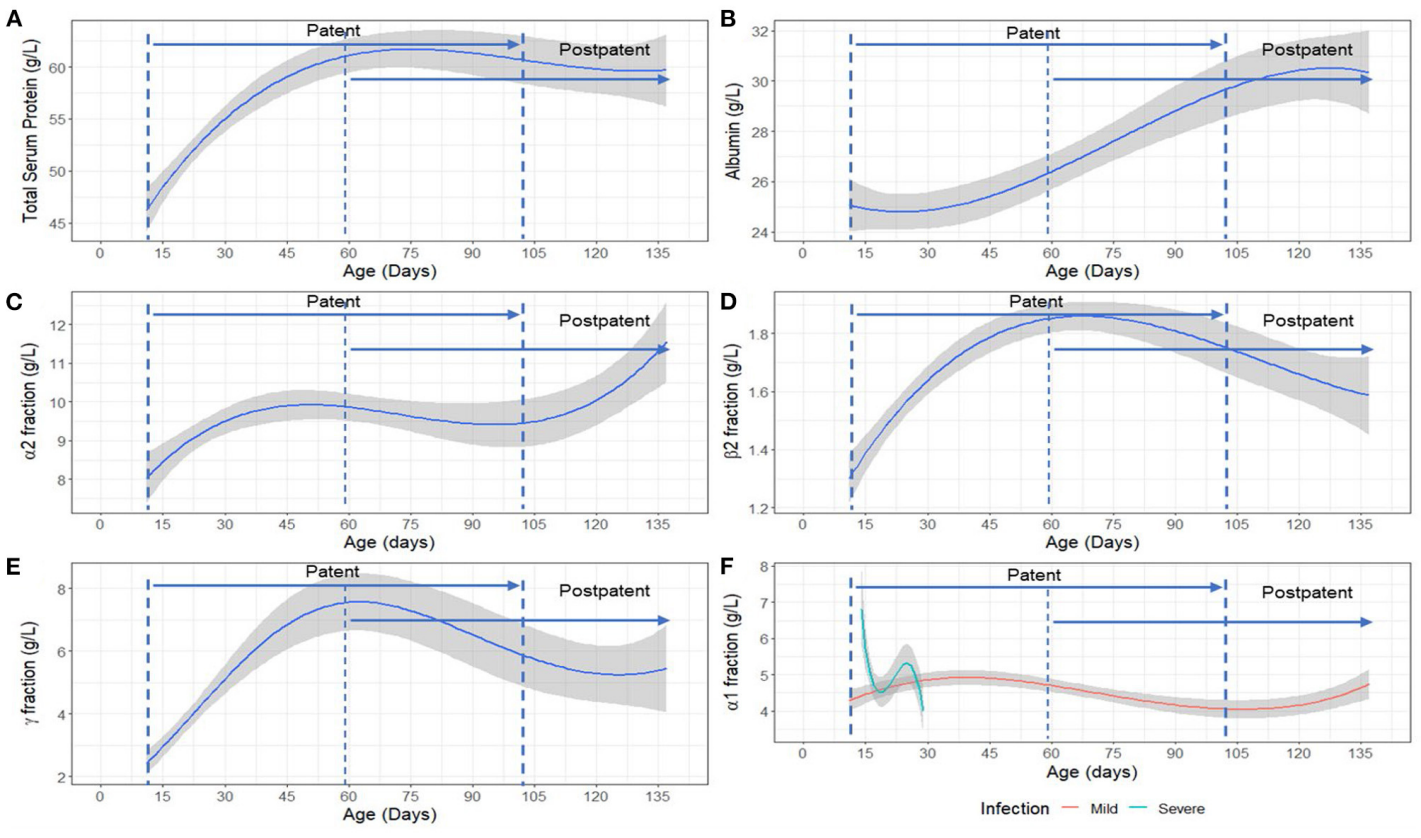
Model estimated relationship between **(A)** Total serum protein, **(B)** Albumin fraction, **(C)** α_2_ fraction, **(D)** β_2_ fraction, **(E)** γ- globulin fraction and age, and **(F)** α_1_ fraction with age and disease severity (mild/severe) in Australian sea lion (*Neophoca cinerea*) pups. Mild: TPP ≥ 60 g/L and PCV > 35%; Severe: TPP < 60 g/L and PCV ≤ 35%. The statistical models for β_2_ and γ-globulins were fitted on a log scale, but model derived back-transformed means (emmeans package R) are presented in figures. The shaded area represents the 95% confidence interval. Patency periods adapted from Marcus et al. (15).

The analysis of SPE data in pups sampled at Seal Bay revealed a significant positive association with age for total serum protein [*F*_(3,50)_ = 26.65, *p* < 0.001], α_2_ [*F*_(3,50)_ = 3.24, *p* < 0.05], β_2_ [*F*_(3,37)_ = 13.00 *p* < 0.001] and γ- globulin fractions [*F*_(3,50)_ = 22.47, *p* < 0.001] (Figure 3). The interaction between age and disease severity was significant for the α_1_ fraction [*F*_(3,32)_ = 6.31, *p* < 0.001], such that changes across time for the α_1_ fraction were greater in animals categorized as having “severe” disease compared to those categorized as “mild” (Figure 3F). In addition, albumin concentration varied significantly with age [*F*_(3,33)_ = 7.36, *p* < 0.001] (Figure 3B). The β_1_ fraction, although not significantly associated with age, was significantly lower [*F*_(1,42)_ = 7.30, *p* < 0.001] in severely diseased animals compared to mildly-diseased pups. Details of all models fitted to acute-phase protein fractions can be found in Appendix B, Table B. 2. Descriptive statistics (mean ± SD) for all the serum protein fractions are provided in Appendix C, Table C. 1.

**Figure 4:**
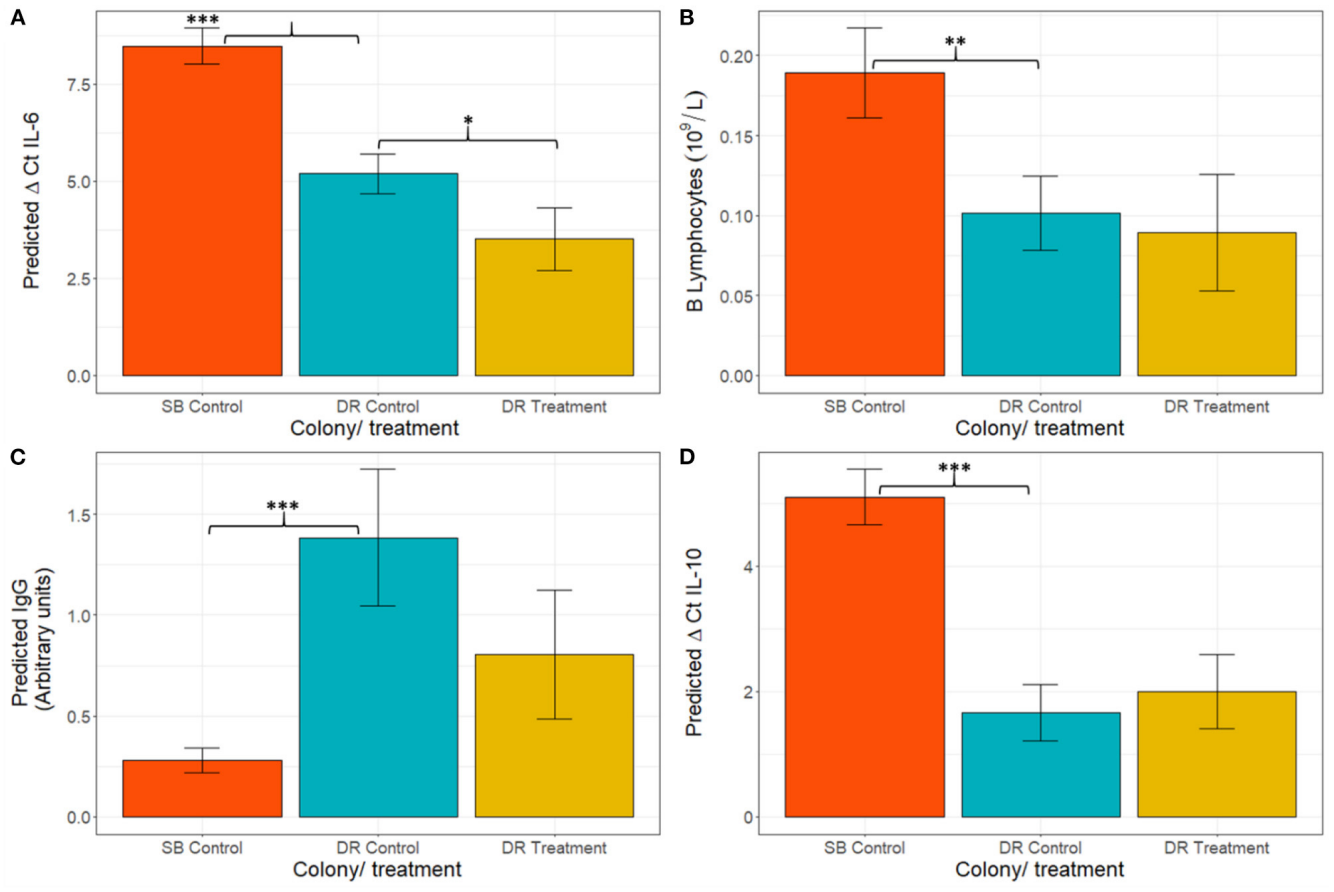
Figure Model estimated mean ± standard error for **(A)** ΔCt IL-6, **(B)** B lymphocytes, **(C)** IgG, and **(D)** ΔCt IL-10. Statistical models for IgG and B lymphocytes were fitted on a log scale, but model derived back-transformed means (emmeans package, R) are presented in figures. Significance codes (*p*): “*” 0.05; “**” 0.01; “***” 0.001. Lower ΔCt indicates greater cytokine expression.

When comparing hookworm intensity between seasons, the multivariable analysis demonstrated significant differences in IgG concentration [*F*_(2,59)_ = 20.38, *p* < 0.001], derived B lymphocyte counts [*F*_(2,30)_ = 10.80, *p* < 0.001], and gene expression of IL-6 [*F*_(2,48)_ = 57.79, *p* < 0.001] and IL-10 [*F*_(2,36)_ = 15.16, *p* < 0.001] between the high hookworm intensity season (Seal Bay) and the low hookworm intensity season (Dangerous Reef, control) (Figure 4). *Post-hoc* analyses revealed that Seal Bay (high-intensity season) pups had lower IgG concentrations (*t* = 5.82, *p* < 0.001) and IL-6 (t = 5.02, *p* < 0.001) and IL-10 (*t* = 5.47, *p* < 0.001) cytokine gene expression, and greater derived B lymphocyte counts (*t* = 2.667, *p* < 0.05) compared to Dangerous Reef (low-intensity season) pups (Figures 4A,C,D, Appendix B, Tables B. 1–3). In addition, the interaction between standard length and colony (SB control and DR control groups) was significant [*F*_(6,53)_ = 3.57, *p* = 0.004] when modeling lysozyme levels, such that changes in lysozyme levels with increasing pup age were greater in the DR control group (Figure 2F).

To further determine the impact of hookworm infection on pup immune development, the immunophenotypes of DR treatment and DR control groups were compared. Treated pups demonstrated greater expression of IL-6 mRNA [lower ΔCt IL-6; *F*_(2,50)_ = 21.70 *p* < 0.001] (Figure 4C) compared to the control group. Although no other immunological measurements where significantly different between groups, there was a trend of lower expression of IFNγ and higher expression of TNFα in the treated group. All models fitted to immunological variables can be found in Appendix B, Table B. 1.

## Discussion

Only a small proportion of wildlife health assessment studies are carried out on threatened species [reviewed in (67)], such as the endangered Australian sea lion. Disease and associated population declines have been reported in some free-living pinnipeds (10, 11, 68–71). These are likely to be the outcome of multi-factorial disease, ecological and anthropogenic impacts, making determination of cause and effect complex (72). However, investigating factors that can increase individual disease susceptibility is essential to understanding the role of disease in regulating wildlife populations (73). Ecoimmunological studies, that is, those that combine clinical pathology measures with biological variables such as age, sex, species ecology and genetic diversity in conjunction with environmental factors such as pathogens, persistent organic pollutants (POPs) and seasonality, are important to evaluate population resilience and potential for disease to impact on population dynamics (3, 41, 74).

This study describes the changes in immune function in neonatal Australian sea lion pups within the context of endemic hookworm infection, the only macroparasite identified in the gastrointestinal tract of pups (15, 75). During the early patent period, a peak in IL-6 mRNA was observed. This was followed by a uniform increase in acute phase proteins, based on lysozyme and SPE, and then B lymphocyte numbers throughout the patent period. This culminated in a Th2-promoted anti-helminth inflammatory responses (elevated B lymphocyte numbers and IgG), around the time where Australian sea lion pups start to naturally eliminate hookworm parasites (15) (75 ± 5 cm standard length; 60 ± 5 days estimated age). Similar trends have been demonstrated in New Zealand sea lions (37), where IgG levels also increased with age; however, the present study shows the broader immune context of these changes. Our results indicate that, as described in killer whales and bottlenose dolphins (76), IL-6 marks the early stages of inflammation, stimulating the acute phase response and activation of B lymphocytes via immunoglobulin class switching and immunoglobulin production (77). The observed delay between IL-6 and subsequent B lymphocyte expansion and IgG production is consistent with the seven to 9 days required by B lymphocytes to become activated, enhance immunoglobulin class switching and produce IgG (78, 79). The age-related changes in SPE profiles are consistent with other findings in canine (*C. familiaris*) pups and bovine calves (31, 38). Acute-phase proteins are a set of non-specific markers from the innate response and have therefore been used to detect early inflammation in domestic and wildlife species as they elevate or decline within 1 and 3 days after inflammatory stimulus or reactive processes (49, 80–83). General trends, and what are considered negative and positive acute-phase proteins (APP) will generally be conserved across species (80), allowing for evaluation and comparisons. In the present study, consistent with other species, such as the Northern elephant seal (*Mirounga angustirostris*) (83), canines and domestic felids (*Felis catus*) (31), albumin was identified as a negative APP, with a declining concentration in the early stages of the acute phase protein response (APPR) and later increasing throughout hookworm patency.

In contrast with humans, where hookworm infections can occur at any stage of life, the timing of hookworm infection in Australian sea lions in the neonatal period to ~2–3 months of age (15, 20) complicates the separation of immunological changes associated with disease from age-related immune changes. However, the differences seen among animals in this study from high- and low-intensity hookworm seasons, and following treatment with ivermectin, indicate that hookworm infection is significantly modulating many of these changes. Our study has demonstrated that lower concentrations of IgG are associated with greater hookworm disease severity, consistent with a finding in South American fur seals that surviving pups had greater IgG concentrations than those pups who died most likely from hookworm infection (16). Although immunoglobulin E has been associated with parasitic infection in several mammalian species, it has not been found in marine mammals (34, 84) and so IgG was chosen in our study as an indicator of plasma cell maturation. Thus, the potential role of IgE in the elimination of hookworm infections in pinnipeds aged 2–3 months of age remains unclear. As hookworm infections are protein-losing enteropathies, it is possible that the lower IgG concentrations observed in Seal Bay high-intensity season pups is related at least in part, to a resource limitation due to greater enteric protein loss associated with high-intensity hookworm infection. On the other hand, IgG differences seen between colonies may also be partially associated with other elements not measured in this study such as lice and microparasitic infections [e.g., *Giardia duodenalis* (85)], skin injuries and abrasions. Unfortunately, due to the limited sample size, we were unable to meaningfully include the presence and intensity of lice as additional variables in our statistical models for this study. However, the broader immunological view provided in the present study and the ability for treatment to reveal causation strongly suggest that this is at least partly due to parasite-induced immune regulation. Lower IgG concentrations were preceded by reduced IL-6 gene expression and apparent retention of B lymphocytes in circulation rather than differentiation into plasma cells in tissue (78, 86) in Seal Bay high-intensity control pups. There was a concurrent trend toward lower IFNγ (Th1 cytokine inhibited by Th2 responses) levels as an effect of treatment in pups from Dangerous Reef (78, 86). Although this immunomodulation could be driven by the presence of other stressors such as pollutants (87–89) or other concurrent but undetected infectious disease interfering with the immunological response and exacerbating a high parasite burden, the effect of treatment on expression of IL-6 suggests that the suppression of IL-6 and the subsequent dampening of the anti-parasitic T-helper 2 response is at least partly hookworm-driven, as is the case in hookworm infections in humans (90, 91). The suppression of IL-6 may also be driving the lower lysozyme activity in the Seal Bay high-intensity season group as IL-6 has a role in the activation of the APPR (80, 81) of which lysozyme is a part. Additionally, IL-6 can induce an upregulation of lysozyme in human polymorphonuclear cells (PMN) (92), which is consistent with the higher levels of neutrophils reported for Australian sea lion pups in Dangerous Reef control compared to those of Seal Bay control (11). The impact of treatment or hookworm infection intensity on other APP, based on SPE, could not be evaluated as these were only examined in pups at Seal Bay. It is likely that the patent stage of hookworm infection acts as a triggering stimulus for the promotion of the APPR (80, 83). Thus, the suppression of IL-6 could also impact the APPR as this cytokine mediates increasing levels of protein electrophoretic fractions as observed in younger canine pups with hookworm infection (31, 93, 94). More investigations of APP are needed, comparing high- and low-intensity seasons to identify if the changes across time seen in this study are related to an actively acquired immune system, differing hookworm infection intensity, or related to other confounding features, for example, cumulative physiological stress due to repeated capture. However, capture method was consistent across the groups, and we therefore don't expect it to be responsible for the differences noted between groups.

The higher gene expression of IL-6 in the Dangerous Reef treatment group suggests that this cytokine might be a more sensitive marker for immune activation than other parameters measured in this study, or clinical observation. Apart from absolute eosinophil counts and red blood cell values, both the present study and Marcus et al. (54) indicated no other significant effect of anthelminthic treatment on immune or clinical parameters, consistent with the administration of the treatment relatively late in the course of infection. A larger effect of treatment would likely have been found if animals had been treated earlier, as shown for clinical parameters in the recent study by Lindsay et al. (20), where ivermectin treatment was given to Australian sea lion pups <2 weeks old. Interleukin 6 has been described previously as a sensitive marker of inflammation in carnivores (49, 95, 96) and other mammalian species of veterinary importance (97–99).

The ecological context can strongly affect the interpretation of immune defenses in the wild. Therefore, the use of a comprehensive multifactorial approach, such as the one described herein, is vital to better understand the complexity of host-parasite interactions. This is the first study exploring cytokine gene expression together with serological measures of immunity as part of an ecoimmunological approach in free-ranging Australian sea lion pups. Several factors, including POPs, have been postulated as metabolic stressors and immunomodulators in humans and wild animals (87–89). Recently, per- and poly-fluorinated alkyl substances (PFAS) used in commercial and industrial applications were identified in the liver of Australian pinnipeds, including in Australian sea lion pups (22). However, their potential effect on disease susceptibility is yet unknown. The identification of sensitive biomarkers for early stages of inflammation and resilience to hookworm infections, and the understanding of their context obtained from this study, will pave the way for future immunological studies in the species and will serve as tools to address the potential effect of pollutants and other anthropogenic stressors on immune responses, to inform management strategies to aid the conservation of this endangered species.

## Data Availability Statement

The raw data supporting the conclusions of this article will be made available by the authors, without undue reservation.

## Ethics Statement

The animal study was reviewed and approved by Government of South Australia Department of Environment, Water and Natural Resources Wildlife Ethics Committee and Scientific Research Permits.

## Author Contributions

M-IM was the primary author of this publication and contributed to the manuscript's study design, analysis, and writing of the manuscript. RG and DH contributed toward the study design, interpretation of findings, and critical revision of the manuscript. AM, RG, and DH also contributed to the sample collection. Under DH's supervision, LB contributed the flow cytometry protocol and data. KS contributed the IgG protocol and data. AC contributed the lysozyme assay protocol and data. PT assisted with guidance on the statistical analyses and contributed with critical revision of the manuscript. All authors contributed to the article and approved the submitted version.

## Funding

This work was supported by the Australian Marine Mammal Centre, Department of the Environment, Australian Government (grant number 09/17) and CONICYT PFCHA through the postgraduate scholarship MAGISTER BECAS CHILE 2017 provided to M-IM (73181623). The funders had no role in study design, data collection and analysis, decision to publish, or manuscript preparation.

## Conflict of Interest

The authors declare that the research was conducted in the absence of any commercial or financial relationships that could be construed as a potential conflict of interest.

## Publisher's Note

All claims expressed in this article are solely those of the authors and do not necessarily represent those of their affiliated organizations, or those of the publisher, the editors and the reviewers. Any product that may be evaluated in this article, or claim that may be made by its manufacturer, is not guaranteed or endorsed by the publisher.
