# Supplementary material for: Developing Immune Profiles of Endangered Australian Sea Lion (Neophoca cinerea) Pups Within the Context of Endemic Hookworm (Uncinaria sanguinis) Infection
Source: Front Vet Sci. 2022 Apr 21;9:824584. doi: 10.3389/fvets.2022.824584 (PMC9069138; doi:10.3389/fvets.2022.824584)
Supplement: Supplementary file 1 [file Data_Sheet_1.docx]

# Appendix A. Laboratory methods for immune-related measures used in the study

## Flow cytometry

Methods follow Lau et al. (2012). In brief, samples stored at -80⁰C in 1x FACS lysing solution (BD Biosciences, San Jose, USA) were thawed in a 37 °C water bath, centrifuged at 1500 x g for 15 minutes and the supernatant removed by pipette. All subsequent centrifugation steps were performed at 800 x g. Cells were resuspended in the remaining fluid, and triplicates distributed into a round-bottomed 96 well plate and then centrifuged at room temperature for 5 min. The penetration of the antibodies to intracellular targets was facilitated by treating cells with 200 μL of 1x FACS Permeabilizing Solution 2 (BD Biosciences, San Jose, USA) in the dark for 15 min. All subsequent wash steps were performed with 1x phosphate-buffered solution (PBS) containing 1 % fetal calf serum (FCS). The cells were washed twice and incubated in the dark for 1 hour at 4 °C in 50 μL volume of PBS containing 10 % FCS and antibodies to optimum concentration (Table A. 1). Cells were then washed twice and centrifuged at room temperature for 5 min. The samples were acquired using a FacsCalibur flow cytometer (Becton Dickinson and Co., North Ryde, Australia). Forward scatter (FSC), and side scatter (SSC) plots were used to gate lymphocytes, and data acquired on compensated fluorescence channels, using FlowJo v8.0 (Tree Star, Inc., Ashland, USA). Post-acquisition analysis was conducted using Flowing Software (Cell Imaging Core, Turku Centre for Biotechnology, Finland; http://flowingsoftware.btk.fi). For statistical analysis, flow cytometric differential counts of lymphocyte subsets were transformed into absolute lymphocyte subset counts (cells x 10^^9^/L) using total lymphocyte count data generated from the same samples for a previous study (Marcus et al., 2015)

Antibody cross-reactivity and optimum concentration were determined by titration in flow cytometry and specificity also evaluated by immunohistochemistry of Australian sea lion lymph nodes. Antibodies against intracellular epitopes of human CD3 (T lymphocytes) and CD79b (B lymphocytes) (Table A. 1) resulted in the labelling of discrete populations of cells in fluorescence plots. The labelling of CD3 positive cells and CD79b positive cells was shown to be mutually exclusive, and CD3 positive cells formed a discrete population of approximately 50 - 70% of lymphocytes (Figure A. 1, A). The B cell antibody marker (CD79b) labelled a small but discrete cell population (Figure A. 1, B).

| Antibody | Species | Target | Conjugation | Concentration |
| --- | --- | --- | --- | --- |
| CD3^a^ | Monoclonal  Rat IgG1 | T cells | PE (LightningLink^®^, Innova Biosciences) | 1/800 |
| CD79b^b^ | Monoclonal  Rat IgG1 | B cells | FITC (LightningLink^®^, Innova Biosciences) | 1/50 |

Table A. 1Staining protocol parameters for flow cytometry


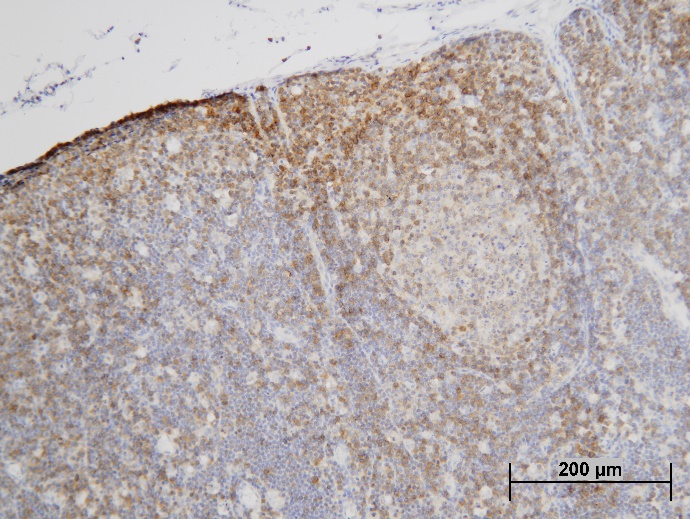
a. CD3 (MCA 1477, AbD serotec); b. CD79b (MCA 2209, AbD serotec).

**B**

**A**

Figure A. 1
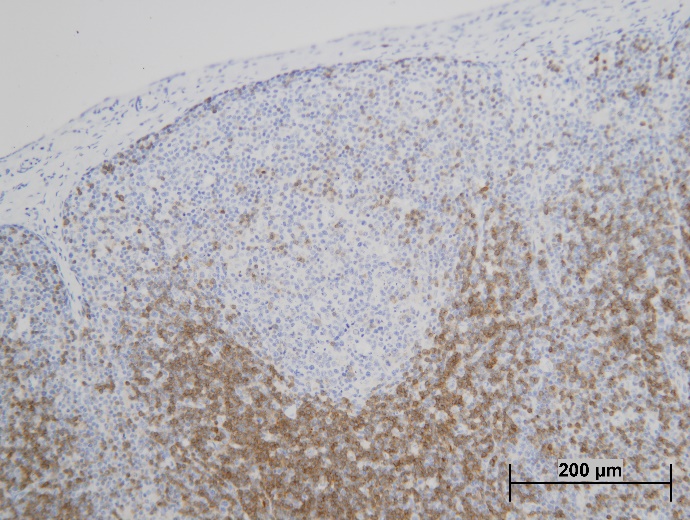
. Photomicrographs of immunostained sections of an Australian sea lion lymph node; chromagen DAB+ (K3467, Dako, Glostrup) (brown) indicates positive labelling against hematoxylin counterstain. A. anti-human CD3 labelled cells illustrating paracortical staining pattern, 40x; B. anti-human CD79b labelled cells in dark zone of cortical follicles, 40x.

## Serum Protein Electrophoresis

Following methods from Gray et al. (2005), 18 µL serum samples were analysed using an automated analyser (SPIFE™ 3000, Helena Laboratories Pty Ltd, Mt Waverley, Australia). Gels were stained with an acid blue stain and scanned using the QuickScan^®^ 2000 densitometer (Helena Laboratories, Australia). The following protein fractions (g/L) were identified: albumin, α_1a_, α_1b_, α_2a_, α_2b_, β_1_, β_2_ and γ-globulin (Figure A. 2). Relative migration distances (Rf) were used to identify each fraction for quantification. Total serum protein concentration (g/L) (TSP) at the time of SPE was determined using a refractometer (TS Meter, Leica Inc., Buffalo, NY, USA). Duplicate readings were obtained for TSP, and the mean of the readings employed for analyses.


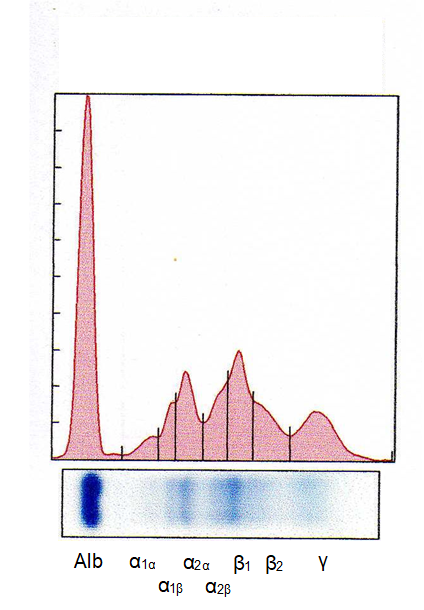


Figure A. 2. Electrophoretogram and representative gel trace for Australian sea lion (*Neophoca cinerea)* serum protein electrophoresis.

## Serum total IgG

A protein A ELISA (Hall et al., 2002) was optimised to laboratory conditions in Australian sea lion sera and then used for relative quantification of total IgG. Serum was diluted to 1:12800 in coating buffer (NaHCO_3_, 9.5 pH), arranged as 100 µL triplicates on a 96 well plate (NUNC MaxiSorp™, Thermo Fisher Scientific Pty. Ltd., Scoresby, Australia), and incubated overnight at room temperature. Following incubation, plates were washed twice with PBS-Tween (PBST). Plates were blocked with 200 µL of 3 % Ovalbumin (Sigma-Aldrich, Castle Hill, Australia) and incubated for 2 hr at 37 °C. Plates were washed twice with PBST, and 100 µL of a 1:500 solution of alkaline phosphatase-conjugated protein A (Merck Millipore, Bayswater, Australia) in PBST was added to each well. Plates were incubated at room temperature for 60 min, washed twice with PBST, followed by a single wash with PBS. A volume of 100 µL Chromagen (p-Nitrophenyl Phosphate, Bio-Rad Laboratories, Hercules, USA) was added to each well and incubated for 7 min at room temperature. The reaction was stopped with 100 µL 0.4M NaOH, and the plates read at 405 nm using a SpectraMax 250 plate reader (Molecular Devices, Sunny Vale, USA). Standards were generated using a serially diluted high-reacting Australian sea lion serum, giving an arbitrary value of 32 units to the maximum standard. The dynamic range of this ELISA spanned five doubling dilutions. Samples that fell outside the dynamic range were re-assayed at revised dilutions, and then values were back-calculated accordingly. Intra assay coefficient of variation (CV) was 13.6%, and the inter-assay CV was < 7.2%.

## Lysozyme assay

A lysoplate assay was adapted from Osserman & Lawlor (1966) to measure Australian sea lion lysozyme activity. Briefly, 1.5% Agarose (Thermo Fisher Scientific Pty. Ltd., Scoresby, Australia) was prepared using Phosphate buffer M/15 (pH 6.3) and *Micrococcus lysodeikticus* (ATCC No. 4698, Sigma-Aldrich Pty. Ltd., Macquarie Park NSW, Australia) to a final concentration of 25 mg of organisms in 100 mL buffered agar. Agarose was poured into Petri dishes to a depth of 4 mm, and 2 mm holes were perforated into the agarose, accommodating 16 sample wells in a Petri dish. Test wells were filled with 10 µL of plasma sample (*n* = 72) and standard dilutions of chicken egg white lysozyme (L6876; Sigma-Aldrich) at 62.5 µg/mL, 12.5 µg/mL, 2.5 µg/mL and 0.5 µg/mL were run with each group of test samples.

Plates were incubated at room temperature for 18 hr, and then clear zones due to bacterial lysis were photographed digitally, and their diameter measured in triplicate using ImageJ Version 1.48 software (Schneider et al., 2012). Mean values were used for statistical analyses and were interpreted using a semilogarithmic plot of measurements of the standards.

## RNA extractions, cDNA and PCR protocols

For RNA extractions, samples in RNAlater™ were thawed at room temperature, centrifuged at 16000 x g for 1 min, and the supernatant discarded from the cell pellet. Total RNA extraction was performed on the cell pellet using the RiboPure™-Blood Kit (Ambion, Carlsbad, CA, USA) according to the manufacturer's instructions, such that the equivalent of 200 µL of whole blood was used per extraction. The RNA concentration and purity were assessed (A_260_/A_280_) using a NanoDrop 1000, Thermo Scientific™ (Waltham, MA, USA) and RNA stored as multiple aliquots at −80 °C for subsequent use.

For analysis, aliquots of RNA were thawed on ice, and two sequential DNase treatments were performed using the RNase-free DNase I (provided in the RNA extraction kit) to eliminate genomic DNA (gDNA). Reverse transcription was performed on 50 - 100 ng of RNA template in a 20 µL reaction with the RevertAid First Strand cDNA Synthesis Kit (Thermo Fisher™, Carlsbad, CA, USA), with a combination (50:50) of random hexamer and oligo(dT)_18_ primers to improve the sensitivity of cDNA synthesis (Ferrante et al., 2018; Gallup, 2011). cDNA was stored as multiple aliquots at –20 °C for subsequent use.

Primers and assay parameters for qPCR were obtained from Meza Cerda et al. (2020). Briefly, amplifications were performed on a CFX96 Real-Time cycler (Biorad, Hercules, CA, USA), following the manufacturer's instructions for the SYBR Green Supermix (SsoAdvanced^TM^ Universal SYBR® Green Supermix, BioRad) and using 4 µL of cDNA template. Primers for IL-6, IL-10, TNFα and GAPDH (Macrogen, Seoul, South Korea) used in this study are summarised in Table A. 2 (Meza Cerda et al., 2020). No-reverse transcription controls (NRT), and no-template controls (NTC), were included in each run. Amplification conditions were 95 °C for 1 min (1 cycle); 95 °C for 10 s and 60 °C for 20 s (40 cycles). A melt curve analysis was generated after each cycling protocol to confirm the absence of non-specific products or primer dimers.

Table A. 2 Characteristics of qPCR primers (IL-6, IL-10, TNFα and GAPDH) used in the study. Conc, primer concentration (nM); Tm, melting temperature (°C).

| Gene | Primers (5'->3') (based on ** marked sequences) | Annealing/Extension (°C, s) | Amplicon size (bp) | Conc (nM) | Primer T_m_ (°C) |
| --- | --- | --- | --- | --- | --- |
| IL-6 | F: CTGCTCCTGGTGATGGCTAC | 60°C, 20 | 147 | 200 | 84.5 |
|  | R: TGCAGAGATTTTGCCGAGGA |  |  |  |  |
| IL-10 | F: CTTTAAGAGTTACCTGGGTTGCC | 60°C, 20 | 97 | 200 | 83.5 |
|  | R: GATGTCTGGGTCGTGGTTCTC |  |  |  |  |
| TNFα | F: GAGCACTGAAAGCATGATCCG | 60°C, 20 | 123 | 200 | 87 |
|  | R: GCGACCAGGAAGAAGGAGAA |  |  |  |  |
| GAPDH | F: TCAACGGATTTGGCCGTATTGG | 60°C, 20 | 90 | 400 | 83.5 |
|  | R: TGAAGGGGTCATTGATGGCG |  |  |  |  |

Primers and assay parameters for the ddPCR workflow were obtained from Meza Cerda et al. (2020), and instruments, reagents and consumables were supplied by Bio-Rad (Bio-Rad, Hercules, CA, USA). Briefly, ddPCR assays were performed using the ddPCR™ Supermix for Probes (no dUTP) in a C1000 Touch™ Thermal Cycler. ddPCR master mix reactions included 10 μL of the Supermix, 1 μL of each primer and probe, 6 μL of cDNA from *N. cinerea* samples and RNase-DNase free water to complete a 20 μL total volume reaction (Table A. 3). Amplification conditions were a 10 min enzyme activation at 95 °C, followed by 40 cycles consisting of denaturation at 95 °C for 30 s and annealing/extension step of 59 °C for 1 min—lastly, an enzyme deactivation period of 10 min at 95 °C and a 4 °C indefinite hold. Droplets were read with a QX200 Droplet Reader, which establishes a threshold between positive and negative droplets based on fluorescence amplitude. Further analysis was performed using the Quanta-Soft Analysis Pro™ software (Bio-Rad). Positive droplets were converted to copy numbers in the PCR mix based on Poisson algorithms (Droplet Digital™ CR Applications Guide, [www.bio-rad.com](http://www.bio-rad.com)).

| Gene Name | Primer Sequences (5'-->3') | Probe Fluorophore | Annealing/ Extension (°C, s) | Amplicon size (Bp) | Conc (nM) |
| --- | --- | --- | --- | --- | --- |
| IFNy | F: AGCTGATTCGAATTCCCGTGA |  | 58, 20 | 95 | 400 |
|  | R: TCTGACTCCTTTTCCGCTTCC |  | 58, 20 |  | 400 |
|  | P: TGCAGGTCCAGCGCAAAGCGATA | FAM | 58, 20 |  | 100 |

Table A. 3 Characteristics of ddPCR primers for (IFNγ) Australian sea lion *(Neophoca cinerea)*  used in the study. Conc, primer/probe concentration (nM).

# References

Ferrante JA, Hunter ME, and Wellehan JFX. 2018. Development and Validation of Quantitative PCR Assays to Measure Cytokine Transcript Levels in the Florida Manatee (*Trichechus manatus latirostris*). *J Wildl Dis* 54:283-294. <https://doi.org/10.7589/2017-06-139>

Gallup J. 2011. qPCR Inhibition and Amplification of Difficult Templates. In: Kennedy S, and Oswald N, eds. *In: PCR troubleshooting and optimisation: the essential guide*. Norfolk, U.K: Caister Academic.

Gray R, Canfield P, and Rogers T. 2005. Serum proteins in the leopard seal, Hydrurga leptonyx, in Prydz Bay, Eastern Antarctica and the coast of NSW, Australia. *Comparative Biochemistry and Physiology Part B: Biochemistry and Molecular Biology* 142:67-78. <https://doi.org/10.1016/j.cbpc.2005.05.016>

Hall AJ, McConnell BJ, and Barker RJ. 2002. The effect of total immunoglobulin levels, mass and condition on the first-year survival of grey seal pups. *Functional Ecology* 16:462–474.

Lau Q, Canfield PJ, and Higgins DP. 2012. Expression and in vitro upregulation of MHCII in koala lymphocytes. *Veterinary Immunology and Immunopathology* 147:35-43. <https://doi.org/10.1016/j.vetimm.2012.04.010>

Marcus A, Higgins DP, and Gray R. 2015. Health assessment of free-ranging endangered Australian sea lion (Neophoca cinerea) pups: effect of haematophagous parasites on haematological parameters. *Comp Biochem Physiol A Mol Integr Physiol* 184:132-143. <https://doi.org/10.1016/j.cbpa.2015.02.017>

Meza Cerda M-I, Gray R, and Higgins DP. 2020. Cytokine RT-qPCR and ddPCR for immunological investigations of the endangered Australian sea lion (Neophoca cinerea) and other mammals. *PeerJ* 8:e10306. 10.7717/peerj.10306

Osserman EF, and Lawlor DP. 1966. Serum and urinary lysozyme (muramidase) in monocytic and monomyelocytic leukemia. *The Journal of experimental medicine* 124:921-952. 10.1084/jem.124.5.921

Schneider CA, Rasband WS, and Eliceiri KW. 2012. NIH Image to ImageJ: 25 years of image analysis. *Nature Methods* 9:671-675. 10.1038/nmeth.2089
