# Supplementary material for: Developing Immune Profiles of Endangered Australian Sea Lion (Neophoca cinerea) Pups Within the Context of Endemic Hookworm (Uncinaria sanguinis) Infection
Source: Front Vet Sci. 2022 Apr 21;9:824584. doi: 10.3389/fvets.2022.824584 (PMC9069138; doi:10.3389/fvets.2022.824584)
Supplement: Supplementary file 2 [file Data_Sheet_2.docx]

# Appendix B

Table B. 1. Final Linear mixed effect models fitted to immune variables from Australian sea lion (*Neophoca cinerea*) pups including treatment group as the main fixed effect.

| **Immunoglobulin G (log(IgG))** | | | | |
| --- | --- | --- | --- | --- |
| *Predictors* | *Estimates* | *CI* | *p* | *df* |
| (Intercept) | -0.44 | -1.51 – 0.62 | 0.415 | 57 |
| Length [1st degree] | 1.04 | -1.96 – 4.05 | 0.495 | 57 |
| Length [2nd degree] | 2.02 | 0.34 – 3.71 | **0.019** | 57 |
| Length [3rd degree] | 0.6 | -1.06 – 2.26 | 0.476 | 57 |
| DR_Ivermectin | -0.22 | -0.91 – 0.48 | 0.543 | 57 |
| SB_Control | -1.18 | -1.67 – -0.68 | **<0.001** | 57 |
| Sex [Male] | -0.44 | -0.81 – -0.07 | **0.02** | 57 |
| Infection [Severe] | -1.29 | -1.98 – -0.59 | **<0.001** | 57 |
| **Random Effects** | | | | |
| σ^2^ | 0.55 | | | |
| τ_00_ _Id_ | 0 | | | |
| N _Id_ | 32 | | | |
| Observations | 67 | | | |
| Marginal R^2^ / Conditional R^2^ | 0.569 / NA | | | |

| **Lysozyme (Lys)** | | | | |
| --- | --- | --- | --- | --- |
| *Predictors* | *Estimates* | *CI* | *p* | *df* |
| (Intercept) | 1.13 | 1.08 – 1.18 | **<0.001** | 57 |
| DR_Ivermectin | -0.13 | -0.23 – -0.03 | **0.008** | 57 |
| SB_Control | -0.07 | -0.13 – -0.00 | **0.048** | 57 |
| **Random Effects** | | | | |
| σ^2^ | 0.01 | | | |
| τ_00_ _Id_ | 0 | | | |
| ICC | 0.1 | | | |
| N _Id_ | 32 | | | |
| Observations | 62 | | | |
| Marginal R^2^ / Conditional R^2^ | 0.117 / 0.207 | | | |

| **∆Ct IL6** | | | | |
| --- | --- | --- | --- | --- |
| *Predictors* | *Estimates* | *CI* | *p* | *df* |
| (Intercept) | 3.36 | 2.08 – 4.64 | **<0.001** | 65 |
| DR_Ivermectin | -0.93 | -2.49 – 0.63 | 0.243 | 65 |
| SB_Control | 3.6 | 2.49 – 4.71 | **<0.001** | 65 |
| ∆Ct TNF | 0.31 | 0.13 – 0.50 | **0.001** | 65 |
| **Random Effects** | | | | |
| σ^2^ | 3.23 | | | |
| τ_00_ _Id_ | 0.22 | | | |
| ICC | 0.07 | | | |
| N _Id_ | 33 | | | |
| Observations | 71 | | | |
| Marginal R^2^ / Conditional R^2^ | 0.654 / 0.676 | | | |

| **∆Ct IL10** | | | | |
| --- | --- | --- | --- | --- |
| *Predictors* | *Estimates* | *CI* | *p* | *df* |
| (Intercept) | 1.48 | 0.56 – 2.40 | **0.002** | 64 |
| DR_Ivermectin | 0.42 | -0.40 – 1.24 | 0.315 | 64 |
| SB_Control | 2.49 | 1.38 – 3.61 | **<0.001** | 64 |
| ∆Ct IL6 | 0.14 | 0.03 – 0.26 | **0.015** | 64 |
| **Random Effects** | | | | |
| σ^2^ | 0.58 | | | |
| τ_00_ _Id_ | 1.75 | | | |
| ICC | 0.75 | | | |
| N _Id_ | 33 | | | |
| Observations | 70 | | | |
| Marginal R^2^ / Conditional R^2^ | 0.514 / 0.879 | | | |

| **log(∆Ct TNF)** | | | | |
| --- | --- | --- | --- | --- |
| *Predictors* | *Estimates* | *CI* | *p* | *df* |
| (Intercept) | 1.36 | 1.02 – 1.70 | **<0.001** | 64 |
| Length [1st degree] | -0.11 | -0.99 – 0.77 | 0.808 | 64 |
| Length [2nd degree] | -0.44 | -0.99 – 0.12 | 0.122 | 64 |
| Length [3rd degree] | -0.35 | -0.84 – 0.15 | 0.169 | 64 |
| ∆Ct IL6 | 0.09 | 0.07 – 0.11 | **<0.001** | 64 |
| **Random Effects** | | | | |
| σ^2^ | 0.07 | | | |
| τ_00_ _Id_ | 0.02 | | | |
| ICC | 0.19 | | | |
| N _Id_ | 33 | | | |
| Observations | 71 | | | |
| Marginal R^2^ / Conditional R^2^ | 0.479 / 0.576 | | | |

| **log(IFNy)** | | | | |
| --- | --- | --- | --- | --- |
| *Predictors* | *Estimates* | *CI* | *p* | *df* |
| (Intercept) | 2.27 | 1.32 – 3.22 | **<0.001** | 53.00 |
| DR_Ivermectin | -0.79 | -1.66 – 0.08 | 0.077 | 53.00 |
| SB_Control | 0.29 | -0.52 – 1.10 | 0.487 | 53.00 |
| Sex [Male] | -0.83 | -1.36 – -0.31 | **0.002** | 53.00 |
| DCt_TNF | 0.17 | 0.06 – 0.29 | **0.004** | 53.00 |
| DCt_IL6 | -0.27 | -0.41 – -0.13 | **<0.001** | 53.00 |
| **Random Effects** | | | | |
| σ^2^ | 0.99 | | | |
| τ_00_ _Id_ | 0.00 | | | |
| N _Id_ | 31 | | | |
| Observations | 61 | | | |
| Marginal R^2^ / Conditional R^2^ | 0.292 / NA | | | |

Table B. 2 Final Linear mixed effect models fitted to Serum Protein Electrophoresis (SPE) data from Australian sea lion (*Neophoca cinerea*) pups sampled at Seal Bay. Age was the main fixed effect.

|  | **Albumin** |  |  |  |
| --- | --- | --- | --- | --- |
| *Predictors* | *Estimates* | *CI* | *p* | *df* |
| (Intercept) | 25.09 | 23.11 – 27.06 | **<0.001** | 44 |
| Age [1st degree] | -1.94 | -8.52 – 4.64 | 0.563 | 44 |
| Age [2nd degree] | 6.68 | 1.11 – 12.25 | **0.019** | 44 |
| Age [3rd degree] | 5.26 | 1.55 – 8.98 | **0.005** | 44 |
| **Random Effects** | | | | |
| σ^2^ | 4.36 | | | |
| τ_00_ _Id_ | 5.33 | | | |
| ICC | 0.55 | | | |
| N _Id_ | 22 | | | |
| Observations | 50 | | | |
| Marginal R^2^ / Conditional R^2^ | 0.255 / 0.665 | | | |

|  | **TSP** |  |  |  |
| --- | --- | --- | --- | --- |
| *Predictors* | *Estimates* | *CI* | *p* | *df* |
| (Intercept) | 49.33 | 44.93 – 53.73 | **<0.001** | 43 |
| Age [1st degree] | 24.57 | 10.60 – 38.55 | **0.001** | 43 |
| Age [2nd degree] | 12.13 | 0.88 – 23.38 | **0.035** | 43 |
| Age [3rd degree] | 13.45 | 5.71 – 21.19 | **0.001** | 43 |
| Infection [Severe] | -6.19 | -10.43 – -1.94 | **0.004** | 43 |
| **Random Effects** | | | | |
| σ^2^ | 23.05 | | | |
| τ_00_ _Id_ | 0 | | | |
| N _Id_ | 22 | | | |
| Observations | 50 | | | |
| Marginal R^2^ / Conditional R^2^ | 0.643 / NA | | | |

|  | **α1a** |  |  |  |
| --- | --- | --- | --- | --- |
| *Predictors* | *Estimates* | *CI* | *p* | *df* |
| (Intercept) | 3.69 | 3.19 – 4.19 | **<0.001** | 44 |
| Age [1st degree] | 0.25 | -1.46 – 1.96 | 0.773 | 44 |
| Age [2nd degree] | -2.39 | -3.83 – -0.95 | **0.001** | 44 |
| Age [3rd degree] | -0.58 | -1.54 – 0.38 | 0.235 | 44 |
| **Random Effects** | | | | |
| σ^2^ | 0.3 | | | |
| τ_00_ _Id_ | 0.27 | | | |
| ICC | 0.48 | | | |
| N _Id_ | 22 | | | |
| Observations | 50 | | | |
| Marginal R^2^ / Conditional R^2^ | 0.233 / 0.598 | | | |

|  | **log(α1b)** |  |  |  |
| --- | --- | --- | --- | --- |
| *Predictors* | *Estimates* | *CI* | *p* | *df* |
| (Intercept) | 0.26 | -0.05 – 0.57 | 0.099 | 38 |
| Age [1st degree] | 0.41 | -0.65 – 1.47 | 0.445 | 38 |
| Age [2nd degree] | 0.21 | -0.64 – 1.06 | 0.625 | 38 |
| Age [3rd degree] | 0.24 | -0.34 – 0.81 | 0.423 | 38 |
| **Random Effects** | | | | |
| σ^2^ | 0.13 | | | |
| τ_00_ _Id_ | 0 | | | |
| N _Id_ | 21 | | | |
| Observations | 44 | | | |
| Marginal R^2^ / Conditional R^2^ | 0.049 / NA | | | |

|  | **α2a** |  |  |  |
| --- | --- | --- | --- | --- |
| *Predictors* | *Estimates* | *CI* | *p* | *df* |
| (Intercept) | 7.84 | 7.49 – 8.18 | **<0.001** | 46 |
| Infection [Severe] | -0.1 | -0.93 – 0.73 | 0.821 | 46 |
| **Random Effects** | | | | |
| σ^2^ | 1.12 | | | |
| τ_00_ _Id_ | 0.1 | | | |
| ICC | 0.08 | | | |
| N _Id_ | 22 | | | |
| Observations | 50 | | | |
| Marginal R^2^ / Conditional R^2^ | 0.001 / 0.080 | | | |

|  | **α2b** |  |  |  |
| --- | --- | --- | --- | --- |
| *Predictors* | *Estimates* | *CI* | *p* | *df* |
| (Intercept) | 0.98 | 0.47 – 1.49 | **<0.001** | 30 |
| Age [1st degree] | 3.84 | 2.15 – 5.52 | **<0.001** | 30 |
| Age [2nd degree] | 0.26 | -1.13 – 1.64 | 0.716 | 30 |
| Age [3rd degree] | 1.64 | 0.75 – 2.53 | **<0.001** | 30 |
| **Random Effects** | | | | |
| σ^2^ | 0.22 | | | |
| τ_00_ _Id_ | 0.17 | | | |
| ICC | 0.44 | | | |
| N _Id_ | 17 | | | |
| Observations | 36 | | | |
| Marginal R^2^ / Conditional R^2^ | 0.412 / 0.669 | | | |

|  | **log(β1)** |  |  |  |
| --- | --- | --- | --- | --- |
| *Predictors* | *Estimates* | *CI* | *p* | *df* |
| (Intercept) | 1.25 | 1.03 – 1.47 | **<0.001** | 43 |
| Age [1st degree] | -0.17 | -0.82 – 0.47 | 0.6 | 43 |
| Age [2nd degree] | 0.54 | -0.01 – 1.08 | 0.053 | 43 |
| Age [3rd degree] | -0.44 | -0.81 – -0.07 | **0.02** | 43 |
| Infection [Severe] | -0.29 | -0.52 – -0.07 | **0.01** | 43 |
| **Random Effects** | | | | |
| σ^2^ | 0.04 | | | |
| τ_00_ _Id_ | 0.04 | | | |
| ICC | 0.52 | | | |
| N _Id_ | 22 | | | |
| Observations | 50 | | | |
| Marginal R^2^ / Conditional R^2^ | 0.226 / 0.630 | | | |

|  | **β2** |  |  |  |
| --- | --- | --- | --- | --- |
| *Predictors* | *Estimates* | *CI* | *p* | *df* |
| (Intercept) | 3.61 | 2.68 – 4.53 | **<0.001** | 44 |
| Age [1st degree] | 4.72 | 1.41 – 8.03 | **0.005** | 44 |
| Age [2nd degree] | 2.88 | 0.12 – 5.64 | **0.041** | 44 |
| Age [3rd degree] | 1.15 | -0.69 – 3.00 | 0.221 | 44 |
| **Random Effects** | | | | |
| σ^2^ | 1.25 | | | |
| τ_00_ _Id_ | 0.33 | | | |
| ICC | 0.21 | | | |
| N _Id_ | 22 | | | |
| Observations | 50 | | | |
| Marginal R^2^ / Conditional R^2^ | 0.364 / 0.498 | | | |

| **log(Gamma globulins)** | | | | |
| --- | --- | --- | --- | --- |
| *Predictors* | *Estimates* | *CI* | *p* | *df* |
| (Intercept) | 1.18 | 0.85 – 1.52 | **<0.001** | 42 |
| Age [1st degree] | 2.16 | 1.16 – 3.16 | **<0.001** | 42 |
| Age [2nd degree] | 0.45 | -0.35 – 1.26 | 0.272 | 42 |
| Age [3rd degree] | 0.79 | 0.23 – 1.34 | **0.005** | 42 |
| Sex [Male] | -0.17 | -0.36 – 0.03 | 0.091 | 42 |
| Infection [Severe] | -0.43 | -0.74 – -0.13 | **0.005** | 42 |
| **Random Effects** | | | | |
| σ^2^ | 0.12 | | | |
| τ_00_ _Id_ | 0 | | | |
| N _Id_ | 22 | | | |
| Observations | 50 | | | |
| Marginal R^2^ / Conditional R^2^ | 0.623 / NA | | | |

Table B. 3 Final Linear mixed effect models fitted to T and B Lymphocytes as measured by flow cytometry in Australian sea lion (*Neophoca cinerea*) pups.

| **log(T)** | | | | | | | | |  |
| --- | --- | --- | --- | --- | --- | --- | --- | --- | --- |
| *Predictors* | *Estimates* | | *CI* | | *p* | | *df* | |  |
| (Intercept) | 1.14 | | 0.79 – 1.50 | | **<0.001** | | 32 | |  |
| Length [1st degree] | -0.09 | | -1.16 – 0.99 | | 0.874 | | 32 | |  |
| Length [2nd degree] | -0.99 | | -1.79 – -0.19 | | **0.015** | | 32 | |  |
| Length [3rd degree] | -0.51 | | -1.09 – 0.07 | | 0.084 | | 32 | |  |
| **Random Effects** | | | | | | | | |  |
| σ^2^ | 0.07 | | | | | | | |  |
| τ_00_ _Id_ | 0.05 | | | | | | | |  |
| ICC | 0.39 | | | | | | | |  |
| N _Id_ | 15 | | | | | | | |  |
| Observations | 38 | | | | | | | |  |
| Marginal R^2^ / Conditional R^2^ | 0.256 / 0.546 | | | | | | | |  |
|  |  | | | | | | | |  |
| **log(B)** | | | | | | | | | |
| *Predictors* | | *Estimates* | | *CI* | | *p* | | *df* | |
| (Intercept) | | -2.48 | | -2.92 – -2.04 | | **<0.001** | | 30 | |
| DR_Ivermectin | | 0.03 | | -0.59 – 0.66 | | 0.924 | | 30 | |
| SB_Control | | 0.56 | | 0.21 – 0.90 | | **0.002** | | 30 | |
| Length [1st degree] | | 0.65 | | -0.70 – 2.01 | | 0.344 | | 30 | |
| Length [2nd degree] | | 0.26 | | -0.75 – 1.27 | | 0.615 | | 30 | |
| Length [3rd degree] | | 0.76 | | -0.01 – 1.52 | | 0.052 | | 30 | |
| **Random Effects** | | | | | | | | | |
| σ^2^ | | 0.14 | | | | | | | |
| τ_00_ _Id_ | | 0 | | | | | | | |
| N _Id_ | | 15 | | | | | | | |
| Observations | | 38 | | | | | | | |
| Marginal R^2^ / Conditional R^2^ | | 0.466 / NA | | | | | | | |
