# Supplementary material for: Developing Immune Profiles of Endangered Australian Sea Lion (Neophoca cinerea) Pups Within the Context of Endemic Hookworm (Uncinaria sanguinis) Infection
Source: Front Vet Sci. 2022 Apr 21;9:824584. doi: 10.3389/fvets.2022.824584 (PMC9069138; doi:10.3389/fvets.2022.824584)
Supplement: Supplementary file 3 [file Data_Sheet_3.docx]

# Appendix C

Table C. 1 Descriptive statistics for serum protein electrophoresis fractions from Australian sea lion pups (*Neophoca cinerea*) in Seal Bay classified by patency periods and disease groups (Mild = TPP ≥ 60g/L and PCV > 35%; Severe = TPP < 60g/L and PCV ≤ 35%) of hookworm disease. (Note - no post-patent pup were classified with severe disease).

|  | **Patent** | | **Post Patent** |
| --- | --- | --- | --- |
|  | **Mild** | **Severe** | **Mild** |
|  | **(N=22)** | **(N=8)** | **(N=19)** |
| **TSP** |  |  |  |
| Mean (SD) | 58.0 (7.48) | 47.9 (2.47) | 64.6 (4.48) |
| **Albumin** |  |  |  |
| Mean (SD) | 25.4 (3.38) | 22.8 (1.42) | 28.4 (2.85) |
| **α** |  |  |  |
| Mean (SD) | 15.6 (1.70) | 14.6 (1.40) | 16.6 (1.50) |
| **α1α** |  |  |  |
| Mean (SD) | 3.5 (0.878) | 3.78 (0.962) | 2.86 (0.567) |
| **a1b** |  |  |  |
| Mean (SD) | 1.6 (0.633) | 1.65 (0.370) | 1.74 (0.579) |
| **α2α** |  |  |  |
| Mean (SD) | 7.8 (1.24) | 7.72 (1.07) | 7.89 (1.06) |
| **α2b** |  |  |  |
| Mean (SD) | 2.3 (0.770) | 1.65 (0.386) | 2.77 (0.825) |
| **β** |  |  |  |
| Mean (SD) | 8.8 (2.06) | 6.64 (0.686) | 9.89 (2.10) |
| **β1** |  |  |  |
| Mean (SD) | 3.5 (0.877) | 3.06 (1.24) | 3.55 (1.20) |
| **β2** |  |  |  |
| Mean (SD) | 5.4 (1.41) | 4.26 (0.788) | 6.37 (1.72) |
| **Log γ-globulin** |  |  |  |
| Mean (SD) | 1.9 (0.466) | 1.09 (0.388) | 2.10 (0.410) |

TPP: Total plasma protein; PCV: Pack cell volume; TSP: Total serum protein.

Note: Additional animals were available for descriptive statistics, which did not have a complete dataset for principal component and multivariable analysis.
